# Dissociation between implicit and explicit gaze perception and their metacognitive representations scales with the degree of autistic traits

**DOI:** 10.1016/j.isci.2026.115602

**Published:** 2026-04-04

**Authors:** Patricia Christian, Ronja Löfström, Arvid Guterstam

**Affiliations:** 1 Karolinska Institutet, Department of Clinical Neuroscience, Stockholm, Sweden

**Keywords:** Behavioral neuroscience, Sensory neuroscience, Cognitive neuroscience

## Abstract

Gaze perception is crucial for inferring others’ attentional state. However, it remains understudied whether implicit (automatic) and explicit (deliberate) gaze perception rely on distinct underlying mechanisms and whether these are affected by autistic traits. We used the same real-life images with an actor being surrounded by objects for two separate tasks: Participants either identified the object the actor was gazing at (explicit task) or were instructed to detect a cued object, with the actor’s gaze being task-irrelevant (implicit task). Our findings demonstrate that implicit and explicit gaze perception rely on dissociable mechanisms, as implicit, but not explicit, attentional shifts, are driven by subtle social cues. Autistic traits selectively affected explicit inference of others’ attentional state as well as the metacognitive representation of these challenges, while implicit gaze perception remained intact. Our findings advance our understanding of the distinct mechanisms underlying gaze perception, which are distinctly affected by autistic traits.

## Highlights

• Implicit and explicit gaze perception rely on dissociable mechanisms
• Automatic, rather than explicit, attention shifts are driven by head-body orientation
• Autistic traits affected explicit, not implicit, inference of social gaze cues
• Autistic traits predicted the level of explicit gaze perception and metacognition

## Graphical abstract

**Figure:**
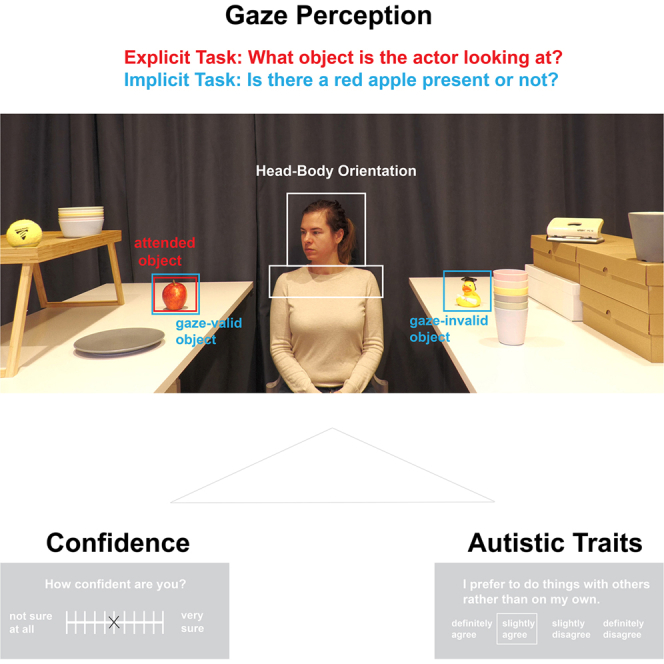

## Introduction

Reconstructing someone else’s attention is central to our ability to understand others’ mental states (mentalization).^1^^,^^2^^,^^3^^,^^4^^,^^5^ While gaze direction provides key information about where someone is looking, inferring their attention requires integrating various social cues, such as head orientation, body posture, and environmental stimuli.^6^^,^^7^ Our brains automatically and implicitly integrate these cues, allowing us to rapidly and reflexively follow others’ gaze without being aware of it.^8^^,^^9^^,^^10^ Crucially, previous findings show that this implicit process facilitates the encoding of another’s attention direction, and not merely another’s gaze direction.^11^^,^^12^^,^^13^^,^^14^ Thus, both implicit and explicit gaze processing contribute to our perception of others’ attention. This observation raises the fundamental question whether these two seemingly distinct systems - implicit versus explicit gaze perception - rely on (at least partially) shared underlying mechanisms.

Autism spectrum disorder (ASD) is characterized by an atypical representation of others’ attention.^15^^,^^16^^,^^17^ Previous findings demonstrate that individuals with ASD show atypical reflexive orienting in the presence of ambiguous social cues, such as when another’s head and body orientation are misaligned.^18^ This finding indicates an impaired ability to integrate social cues to infer others’ attention in an implicit manner. In explicit tasks, individuals with ASD also face challenges in determining others’ gaze targets^19^^,^^20^^,^^21^^,^^22^^,^^23^ and show atypical gaze patterns when trying to determine others’ attentional focus.^21^^,^^24^^,^^25^ Together, these findings suggest that both implicit and explicit gaze perception are affected in ASD. Nevertheless, it has been debated whether social cognitive difficulties in autistic adults are restricted to implicit interpretations of social cues,^26^ while explicit social reasoning skills remain intact, possibly due to compensatory learning.^27^^,^^28^ It remains unclear whether the atypical integration of social cues in autism selectively affects implicit gaze processing or may influence explicit gaze perception ability as well. Addressing this open question may inform us about the relationship between implicit and explicit gaze perception mechanisms in ASD.

Moreover, recent findings suggest that while individuals with ASD experience challenges in inferring others’ minds, they also show a diminished ability to accurately monitor these challenges.^29^^,^^30^ This ability is referred to as metacognitive sensitivity.^31^^,^^32^ It has been proposed that difficulties in representing one’s own mental states (metacognition) may adversely affect the ability to represent others’ mental states (ToM), indicating a shared underlying mechanism that could be affected in autism.^33^^,^^34^ Indeed, recent work has demonstrated that individuals with ASD show alterations in understanding others’ minds, while at the same time showing reduced metacognitive awareness of these alterations.^35^^,^^36^ Given the intimate relationship between social gaze perception and ToM, these findings raise the question of whether autistic traits are associated with decreased metacognitive sensitivity in implicit and explicit gaze perception.

**Figure 1:**
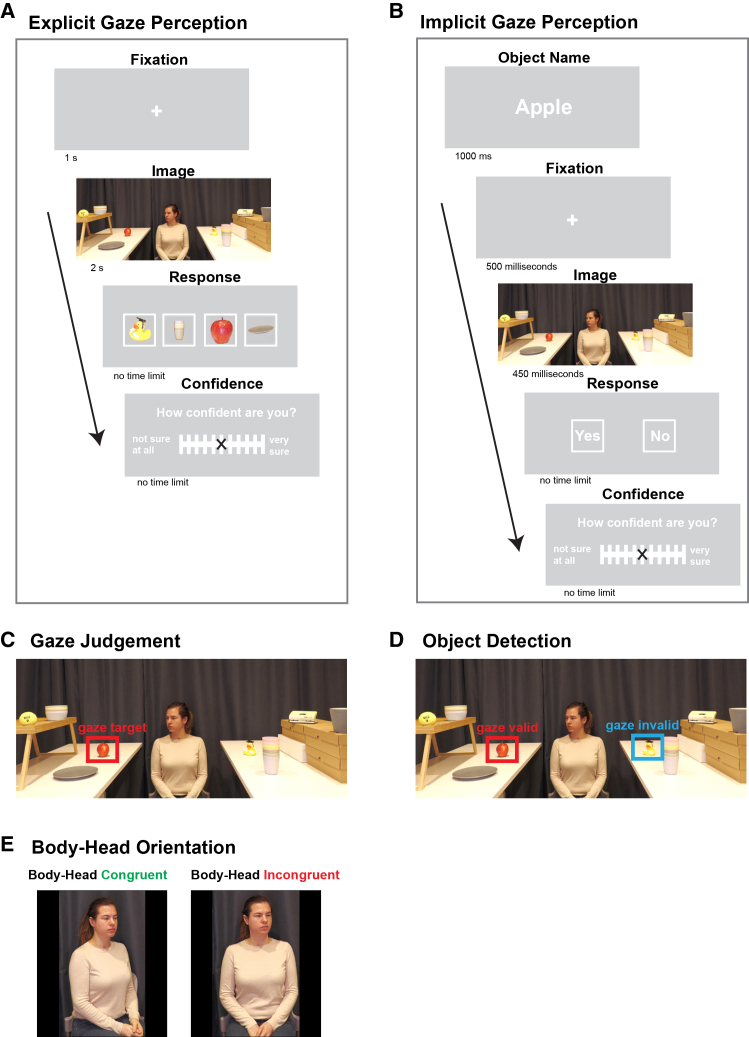
Behavioral paradigm Participants performed two separate tasks in which the same real-life images were presented. Each image showed an actor sitting between two tables with four different objects displayed on each table, gazing at only one of these objects. (A) Explicit gaze perception. Participants were instructed to identify the object that the actor was gazing at (gaze judgment task). In each trial, after a 1-s fixation period, the image was presented for 2 s. Participants then performed a forced-choice task, indicating which object the actor was gazing at. Afterward, participants rated their confidence level in their response using an 11-point Likert scale. (B) Implicit gaze perception. Participants’ task was to detect whether a cued object in the image was present (object detection task). In each trial, the name of an object was displayed for 1 s. After a 500-ms fixation period, the image was presented for 450 ms, after which participants indicated whether the cued object was present (yes) or absent (no) in the image, followed by a confidence rating. (C and D) Example of a real-life image for both tasks. Gaze judgment task (C): In each image, there was only one object the actor gazed at out of four possible objects displayed on the gazed-at side. Object detection task (D): The object that participants were instructed to detect was only present in half of all trials. In these trials, the cued object was either located where the actor was gazing (gaze valid trials) or on the opposite side (gaze invalid trials). (E) Body-head orientation. Half of the images displayed the actor’s head and body both oriented 35° to one side (body-head congruent), while in the other half the actor’s body faced forward while the head was turned 35° to the side (body-head incongruent).

To address these open questions, we examined implicit and explicit gaze processing and metacognitive abilities in 64 neurotypical adults, using autistic traits as a proxy measure of autism.^37^ The experiment involved two tasks, both using the same real-life images of actors gazing at laterally presented target objects. The orientation of the actor’s head with respect to the body was either “congruent” (both head and body turned to the side) or “incongruent” (head turned but body facing straight). The relative head-and-body orientation is an established manipulation of social cues where previous studies have shown that an incongruent orientation, which signifies that something of interest to the side has caught the actor’s attention, facilitates attention orienting in neurotypical but not in autistic subjects.^13^^,^^18^ In the explicit gaze perception task, participants were instructed to identify which object the actor was attending to.^20^^,^^25^ In the implicit gaze perception task, participants performed a visual object detection task in which the actor’s gaze direction and body posture were task-irrelevant.^38^ Our analyses focused on response accuracy. Both tasks were matched in terms of difficulty, and after each trial, participants rated their confidence in their response on a continuous rating scale (Figure 1). To quantify participants’ autistic traits, we used the Autism-Spectrum Quotient (AQ) questionnaire, which is a widely used measure in both neurotypical and autistic populations.^37^

We made four predictions. Based on prior evidence, we hypothesized that incongruent body-head orientation, signaling the saliency of gaze targets, would modulate performance in the implicit but not in explicit gaze perception task, given that implicit gaze-cueing and explicit gaze perception operate on dramatically different timescales (ms versus s). Thus, we hypothesized that subtle social cues would facilitate implicit perception of others’ attention direction, while explicit processes rely on distinct mechanisms operating at a slower timescale.

Second, we predicted that participants who are better at automatically integrating social gaze cues in the implicit task would demonstrate higher accuracy in the explicit gaze perception task, indicating a more efficient general machinery for interpreting visual cues relevant for reconstructing others’ attentional direction.

Third, we predicted that participants with stronger autistic traits would be affected in integrating implicit social cues conveying key information about others’ attention direction (Ashwin et al. 2015), reflected in decreased performance in the implicit task. In line with prior evidence showing that autistic individuals are less accurate at determining others’ gaze targets,^20^ we predicted that participants with higher autistic traits would also show decreased performance in the explicit gaze perception task.

Fourth, we tested whether participants’ ability to accurately represent these perceptual alterations (metacognitive sensitivity) in implicit and explicit gaze processing varied as a function of autistic traits. We predicted that autistic traits would be associated with reduced metacognitive sensitivity in both implicit and explicit gaze perception, thus impacting gaze perception itself as well as the metacognitive sensitivity to these alterations.

## Results

### Implicit and explicit gaze perception rely on distinct underlying mechanisms

**Figure 2:**
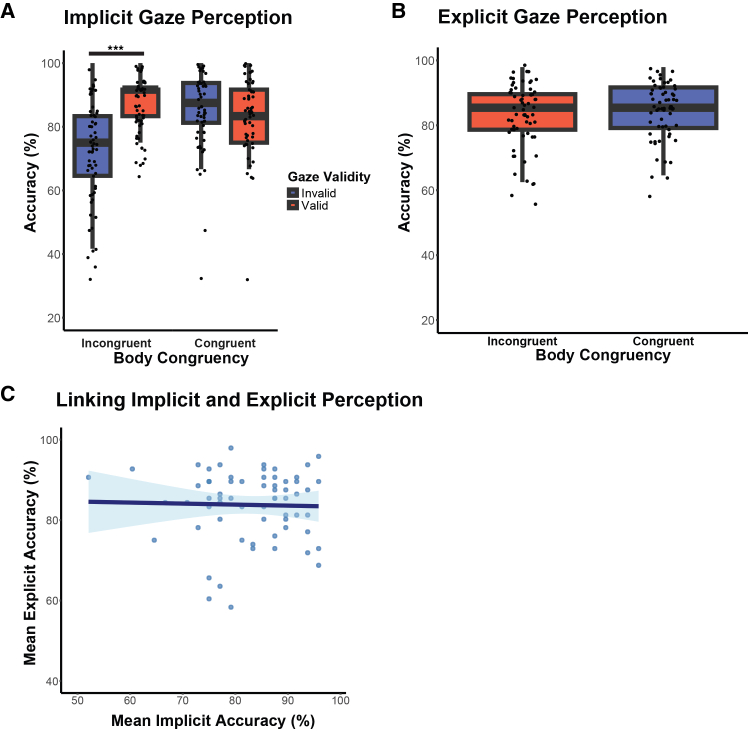
Behavioral results of explicit and implicit gaze perception (A) Participants’ ability to detect the cued object in the four experimental conditions of the implicit gaze perception task. Participants were more accurate when the actor’s gaze was directed toward the cued object (gaze validity: *β* = 0.96, *z* = 6.32, *p* < 0.001), but only when the relative orientation of the actor’s head and body was incongruent (gaze validity x body-head congruency: *β* = −1.04, *z* = - 4.42, *p* < 0.001). Boxplots illustrate median and interquartile range (25th to 75th percentiles) of accuracy scores (%). Individual data points represent each subject’s mean accuracy (%). (B) Participants’ ability to accurately determine the actor’s gaze target in the two experimental conditions of the explicit gaze perception task. In contrast to the implicit task, body-head congruency did not significantly influence participants’ ability to determine others’ attention target (body-head congruency: *β* = 0.07, *z* = 0.94, *p* = 0.35). Note that all trials in the explicit task are gaze valid. (C). Participants’ performance in implicit gaze perception (sensitivity to salient gaze cues) did not significantly predict accuracy in explicit gaze perception (*β* = 0.06, *z* = 0.74, *p* = 0.46). For graphical illustration, we plotted a linear regression on mean accuracy (%) for implicit and explicit gaze perception with 95% confidence interval (CI). Individual data points represent each subject’s mean accuracy (%) for implicit and explicit gaze perception, respectively. ∗*p* < 0.05, ∗∗*p* < 0.01, and ∗∗∗*p* < 0.001.

Figure 2 shows the behavioral results of the implicit and explicit gaze perception tasks. In the implicit task, participants had a mean accuracy of 83% (standard error [SE] = 1,140%) in trials where the cued object was present in the image (i.e., 50% of all trials), indicating a moderate level of task difficulty. Task accuracy over trials in which the object was not present in the image was 95,1% (SE = 0.46%). The overall task accuracy over all trials was 88,9% (SE = 0.64%).

Here, we tested the first hypothesis that head-body congruency would modulate performance in implicit, but not explicit gaze perception, reflecting automatic attention shifts based on the perceived saliency of body-head orientation. To test these predictions, we calculated GLMM accuracy models with the fixed-effect predictors gaze validity, body-head congruency, and their interaction terms for implicit and explicit gaze perception, respectively. Our results on implicit GLMM show that participants were significantly more accurate in detecting cued objects when the actor’s gaze was valid rather than invalid (main effect of gaze validity: *β* = 0.96, *z* = 6.32, *p* < 0.001), suggesting the participants effectively oriented their spatial attention to the actor’s gaze. Crucially, our results demonstrate a significant interaction effect of gaze validity × body-head congruency (*β* = −1.04, *z* = −4.42, *p* < 0.001, see Figure 2A and Table 1), confirming our prediction that people integrate social cues, such as gaze, head, and body orientation, when automatically inferring others’ attention. Further, we calculated post hoc GLMM separately for body-head congruent and body-head incongruent trials to examine how the interplay between gaze, head, and body orientation influences implicit gaze perception. In line with a previous experiment (Ashwin et al. 2015) our results show higher object detection accuracy for gaze-valid versus gaze-invalid trials in the body-head incongruent (gaze validity: *β* = 0.94, *z* = 6.13, *p* < 0.001) but not in the body-head congruent condition (gaze validity: *β* = −0.08, *z* = −0.49, *p* = 0.63). While previous findings indicate that body-head incongruence enhances the saliency of others’ gaze cues,^13^ our results suggest that participants more strongly shifted their attention toward gazed-at objects when others’ heads and bodies were not aligned with each other, facilitating automatic inference of others’ attention.

**Table 1:** Implicit gaze perception accuracy

Predictor	Estimate (SEM)	*z*	*p*
Intercept	1.01 (0.11)	9.56	<0.001∗∗∗
gaze validity	0.96 (0.15)	6.32	<0.001∗∗∗
body-head congruency	0.99 (0.16)	6.28	<0.001∗∗∗
gaze validity × body-head congruency	−1.04 (0.23)	−4.42	<0.001∗∗∗

Table reports GLMM results of implicit gaze perception task: Accuracy was regressed on the fixed-effect predictors gaze validity (0 = invalid gaze cue, 1 = valid gaze cue), body-head congruency (0 = incongruent, 1 = congruent), and their interaction. The table presents fixed-effect parameter estimates *β* (with standard error of the mean, SEM, in brackets), *z*-statistic, and *p*-value. ∗*p* < 0.05, ∗∗*p* < 0.01, and ∗∗∗*p* < 0.001.

In the explicit task, participants had a mean accuracy of 84% (standard error [SE] = 0.01%) in correctly identifying the actor’s gaze target, indicating a task difficulty comparable with the implicit task. There was no significant effect of body-head congruency on explicit, deliberate gaze perception (*β* = 0.07, *z* = 0.94, *p* = 0.35, Figure 2B and Table 2). This finding was further supported by a post hoc *t* test, which tested whether individual coefficients extracted from the implicit and explicit GLMM models are different from each other. The results suggest that the observed effect of body-head congruency in the implicit model significantly differed from the effect on explicit gaze perception (Mean_Implicit_ = −1.04, SE_Implicit_ = 0.03, versus Mean_Explicit_ = 0.01, SE_Explicit_ = 0.01; t_64_ = 38.30, *p* < 0.001). To sum up, these findings suggest that the integration of social perceptual information related to gaze direction, head position, and body orientation of others selectively affects processes related to reflexive orienting to others’ attention direction, but not conscious, explicit gaze perception.

**Table 2:** Explicit gaze perception accuracy

Predictor	Estimate (SEM)	*z*	*p*
Intercept	1.72 (0.09)	18.50	<0.001∗∗∗
body-head congruency	0.07 (0.08)	0.94	0.35

Table reports GLMM results of explicit gaze perception task: Accuracy was regressed on the fixed-effect predictor body-head congruency. The table presents fixed-effect parameter estimates *β* (with standard error of the mean, SEM, in brackets), *z*-statistic, and *p*-value. ∗*p* < 0.05, ∗∗*p* < 0.01, and ∗∗∗*p* < 0.001.

Nevertheless, it remained unclear whether implicit processing of subtle social cues might facilitate explicit inference of others’ attention. In the next step, we tested the second prediction that implicit gaze perception accuracy modulates explicit inferences of others’ gaze. Here, we extracted individual fixed-effect estimates of the gaze validity × body-head congruency interaction from the implicit GLMM and included them as a predictor in the explicit GLMM. Contrary to our prediction, individual differences in implicit sensitivity to gaze saliency did not significantly predict accuracy in the explicit gaze perception task (*β* = 0.06, *z* = 0.74, *p* = 0.46; Figure 2C).

To sum up, these findings suggest that the integration of social perceptual information related to gaze direction, head position, and body orientation of others selectively affects processes related to reflexive orienting to others’ attention direction, but not conscious, explicit gaze perception. Taken together, this dissociation provides evidence for at least partially distinct underlying mechanisms underlying implicit and explicit processing of social gaze cues.

### Autistic traits affect explicit but not implicit gaze perception

Here, we tested the third prediction that participants with stronger autistic traits would show decreased performance in the explicit gaze perception task as well as an impaired integration of social cues for orienting to the actor’s attention in the implicit task (while showing an intact general gaze cueing ability). To test these predictions, we included AQ scores in the GLMM accuracy models for implicit and explicit gaze perception, respectively.

**Figure 3:**
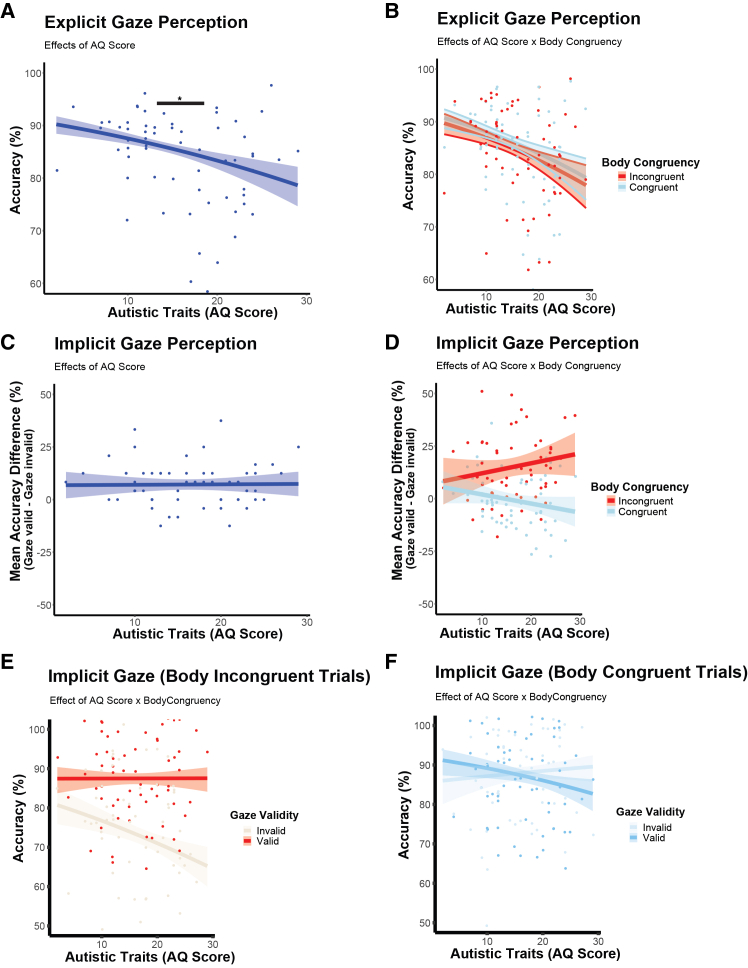
Results of explicit and implicit gaze perception depending on autistic traits (A) Individuals with higher autistic traits were worse at determining the actor’s gaze target in the explicit gaze perception task (AQ score: *β* = −0.21, *z* = −2.4, *p* = 0.02). (B) As expected, this effect was not modulated by body-head orientation (body-head congruency x AQ score: *β* = 0.01, *z* = 0.14, *p* = 0.89). (C) In contrast, participants’ ability to detect cued objects in the implicit gaze perception task was not significantly affected by autistic traits (AQ score: *β* = −0.16, *z* = −1.45, *p* = 0.15). (D) This effect was not significantly modulated by body-head orientation (gaze validity x body-head congruency x AQ score, β = −0.38, z = −1,70, *p* = 0.09). For the graphical presentation of the interaction effects between body-head congruency and gaze validity on accuracy, results are shown separately for body-incongruent (E) and body-congruent trials (F). Explicit gaze perception: Logistic curves plot the relationship between a binary dependent variable (accuracy in %) and a continuous predictor (AQ scores) with 95% CI. Implicit gaze perception: Logistic curves plot the relationship between the dependent variable mean accuracy difference (mean accuracy gaze valid - mean accuracy gaze invalid in %) and AQ score with 95% CI. Individual data points are plotted to represent each subject’s mean accuracy (%). ∗*p* < 0.05, ∗∗*p* < 0.01, and ∗∗∗*p* < 0.001.

In the explicit gaze perception task, the results revealed that participants with higher autistic traits were less accurate in inferring others’ gaze direction (main effect of AQ score: *β* = −0.21, *z* = −2.4, *p* = 0.02, Figure 3A and Table 3), in line with our prediction. To further explore the potential effects of body-head congruency, we also included the predictor of body-head congruency in the model. As expected, body-head congruency did not modulate task performance as a function of autistic traits in the explicit task (body-head congruency x AQ score: *β* = 0.01, *z* = 0.14, *p* = 0.89, see Figure 3B). This effect was not moderated by gender (*β* = −0.02, *z* = −0.12, *p* = 0.90).

**Table 3:** Effects of autistic traits on explicit gaze perception

Predictor	Estimate (SEM)	*z*	*p*
Intercept	1.71 (0.09)	18.86	<0.001∗∗∗
body-head congruency	0.07 (0.08)	0.90	0.37
AQ score	−0.20 (0.10)	−2.12	0.034∗
body-head congruency× AQ score	0.02 (0.08)	0.14	0.90

Table reports GLMM results of explicit gaze perception task: Accuracy was regressed on the fixed-effect predictors body-head congruency, AQ score (continuous scale), and their interaction term. The table presents fixed-effect parameter estimates *β* (with standard error of the mean, SEM, in brackets), *z*-statistic, and *p*-value. ∗*p* < 0.05, ∗∗*p* < 0.01, and ∗∗∗*p* < 0.001.

In the implicit gaze perception task, autistic traits did not significantly impact task performance (AQ score: *β* = −0.16, *z* = −1.45, *p* = 0.15; Figure 3C and Table 4). Notably, participants with higher AQ traits did not significantly differ from those with lower AQ traits in their ability to detect objects based on body orientation and gaze validity (gaze validity x body-head congruency x AQ score, *β* = −0.38, *z* = −1,70, *p* = 0.09, see Figures 3D–3F and Table 2). These results suggest that, in contrast to our initial hypothesis, participants with high autistic traits were able to integrate social information related to body and head orientation to orient attention to others’ gaze to a similar degree as individuals with low autistic traits.

**Table 4:** Effects of autistic traits on implicit gaze perception

Predictor	Estimate (SEM)	*z*	*p*
Intercept	0.98 (0.09)	10.47	<0.001∗∗∗
gaze validity	0.99 (0.15)	6.69	<0.001∗∗∗
body-head congruency	0.99 (0.16)	6.25	<0.001∗∗∗
AQ score	−0.16 (0.11)	−1.45	0.15
gaze validity × body-head congruency	−1.05 (0.23)	−4.60	<0.001∗∗∗
gaze validity × AQ score	0.16 (0.15)	1.08	0.28
body-head congruency× AQ score	0.25 (0.16)	1.58	0.12
gaze validity × body-head congruency × AQ score	−0.38 (0.23)	−1.70	0.09

Table reports GLMM results of implicit gaze perception task: Accuracy was regressed on the fixed-effect predictors gaze validity, body-head congruency, AQ score, and their interaction. The table presents fixed-effect parameter estimates *β* (with standard error of the mean, SEM, in brackets), *z*-statistic, and *p*-value. ∗*p* < 0.05, ∗∗*p* < 0.01, and ∗∗∗*p* < 0.001.

**Figure 4:**
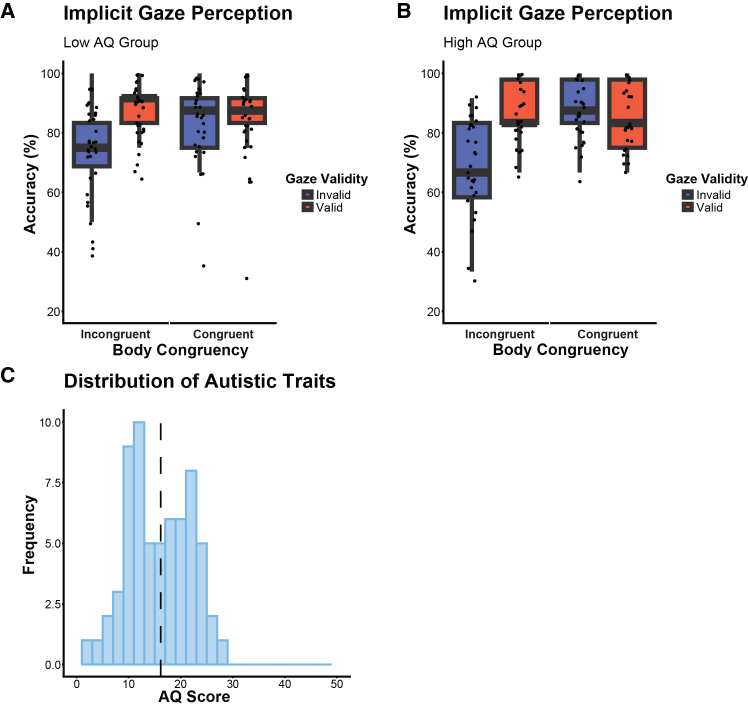
Results of exploratory analysis of AQ group effects (low vs. high AQ) on implicit gaze perception (A and B) Individuals with higher autistic traits showed no significant differences in contrast to lower autistic traits in determining the actor’s gaze target in the explicit gaze perception task (AQ score: *β* = 0.42, *z* = 0.89, *p* = 0.37). Boxplots illustrate accuracy scores as percentages, showing the median and interquartile range (25th to 75th percentiles), with individual data points plotted to represent each subject’s mean accuracy (%) for (A) low AQ group and (B) high AQ Group. (C) Graphical illustration of the distribution of individual AQ scores for the full sample (*N* = 64; range = 2–27). The dotted line indicates the sample mean AQ score (*M* = 16.1, *SD* = 6.08). None of our AQ scores were above the clinical cut-off of 32.

Additionally, to characterize differences between lower and higher autistic traits on task performance in implicit gaze perception, we run an exploratory analysis in which AQ scores were median-split (median AQ score = 16) into two groups (low, high AQ). We calculated a GLMM including gaze validity, body-head congruency, and AQ group. This analysis revealed no significant main effect of AQ group (*β* = 0.32, *z* = 1.5, *p* = 0.13) and no significant interaction effects involving AQ group, gaze validity, or body-head congruency (gaze validity × AQ group: *β* = −0.22, *z* = −0.73, *p* = 0.46; body-head congruency × AQ group: *β* = −0.41, *z* = −1.30, *p* = 0.19; gaze validity × body-head congruency × AQ group: *β* = 0.42, *z* = 0.89, *p* = 0.37, see Figures 4A and 4B, and Table 5), These non-significant group-level results indicate that participants with higher AQ traits did not differ from those with lower AQ traits in their ability to integrate subtle social cues from body and head orientation to implicitly interpret others’ attention. We also checked for gender-related differences, including gender in the GLMM, but the results show no significant effects on gender differences (*β* = 0.07, *z* = 0.13, *p* = 0.90).

**Table 5:** Effects of autistic traits (AQ group) on implicit gaze perception

Predictor	Estimate (SEM)	*z*	*p*
Intercept	0.85 (0.15)	5.79	<0.001∗∗∗
gaze validity	1.08 (0.22)	5.03	<0.001∗∗∗
body-head congruency	1.20 (0.21)	5.54	<0.001∗∗∗
AQ group	0.32 (0.21)	1.50	0.13
gaze validity × body-head congruency	−1.25 (0.34)	−3.68	<0.001∗∗∗
gaze validity × AQ group	−0.22 (0.30)	−0.73	0.46
body-head congruency× AQ group	−0.41 (0.31)	0.31	0.19
gaze validity × body-head congruency × AQ group	0.42 (0.47)	0.47	0.37

Table reports GLMM results of implicit gaze perception task: Accuracy was regressed on the fixed-effect predictors gaze validity, body-head congruency, AQ group, and their interaction. The table presents fixed-effect parameter estimates *β* (with standard error of the mean, SEM, in brackets), *z*-statistic, and *p*-value. ∗*p* < 0.05, ∗∗*p* < 0.01, and ∗∗∗*p* < 0.001.

### Metacognitive sensitivity scales with the degree of autistic traits in explicit but not implicit gaze perception

**Figure 5:**
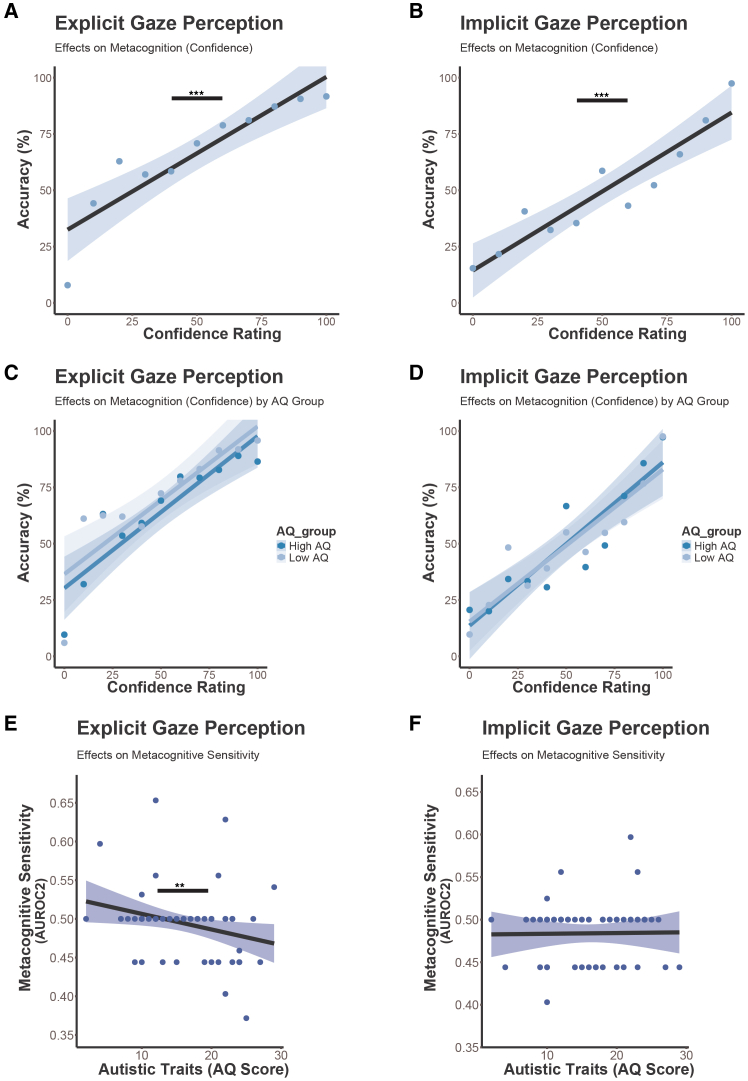
Results of explicit and implicit gaze perception on metacognition depending on autistic traits (A–D) Results of LMER models on confidence ratings: (A) Overall, participants’ accuracy in the task was predictive of their confidence ratings in the explicit (accuracy: β = 1.17, z = 16.86, *p* < 0.001) and (B) implicit task (accuracy: β = 1.66, z = 23.06, *p* < 0.001), suggesting participants could accurately relate performance to confidence, highlighting metacognitive awareness. (C) This was not modulated by autistic traits in the explicit (accuracy × AQ scores: β = −0.02, z = −0.27, *p* = 0.79) and (D) implicit task (accuracy × AQ scores: β = 0.07, z = 0.92, *p* = 0.37) suggesting that even though participants with higher autistic traits showed lower accuracies in explicit gaze perception, this was not reflected in their confidence ratings. We plotted a regression line to illustrate the relationship between a continuous dependent variable (confidence rating) and a binary predictor (accuracy in %) with 95% CI. For graphical illustration we median-splitted AQ scores to differentiate between low and high autistic traits (AQ group). (E and F) Results on metacognitive sensitivity based on AUROC2: (E) Our findings show that individuals with higher autistic traits had more difficulties to accurately monitor their task difficulties in the explicit task (*p* < 0.01) suggesting that subjects with higher autistic traits showed deficits in task accuracy and reduced metacognitive sensitivity. (F) In contrast, autistic traits did not affect metacognitive sensitivity in the implicit task (*p =* 0.45). We plotted a regression to illustrate the correlation between AUROC2 scores and autistic traits (AQ scores) with 95% CI. Individual data points are plotted to represent each individual AUROC2 score. ∗*p* < 0.05, ∗∗*p* < 0.01, and ∗∗∗*p* < 0.001.

Finally, we asked whether metacognitive sensitivity in implicit and explicit gaze processing varied as a function of autistic traits. First, we tested whether autistic traits modulate confidence ratings in the implicit and explicit gaze perception task. Our result show that higher accuracy significantly predicted higher confidence ratings in the explicit task (accuracy: *β* = 1.18, *z* = 16.95, *p* < 0.001, see Figure 5A and Table 6) and in the implicit task (accuracy: *β* = 1.65, *z* = 22.10, *p* < 0.001, see Figure 5B and Table 6), suggesting that participants could accurately evaluate and represent their own performance in both tasks. However, in both tasks there were no significant main effects of AQ score (explicit task: *β* = −0.06, *z* = −0.65, *p =* 0.52; implicit task: *β* = −0.07, *z* = −0.83, *p* = 0.42) or significant accuracy × AQ scores interaction effects (explicit task: *β* = −0.01, *z* = −0.21, *p =* 0.84, Fic 5C, Table 6; implicit task: *β* = 0.07, *z* = 0.93, *p* = 0.36; Figure 5D and Table 6), demonstrating that even though participants with higher autistic traits showed lower accuracies in explicit gaze perception, this was not reflected in their confidence ratings.

**Table 6:** Effects of autistic traits on confidence

Predictor	Estimate (SEM)	t	df	*p*
**Implicit Gaze Perception**				

Intercept	−1.38 (0.08)	58.76	−17.38	<0.001∗∗∗
Accuracy	1.66 (0.07)	57.48	23.06	<0.001∗∗∗
AQ score	−0.06 (0.08)	57.23	−0.80	0.43
Accuracy x AQ score	0.07 (0.07)	47.03	0.92	0.37

**Explicit Gaze Perception**				

Intercept	−1.00 (0.09)	61.10	−11.48	<0.001∗∗∗
Accuracy	1.17 (0.07)	63.48	16.86	<0.001∗∗∗
AQ score	−0.05 (0.09)	52.99	−0.63	0.53
Accuracy x AQ score	−0.02 (0.07)	63.96	−0.27	0.79

Tables reports LMER results of implicit and explicit gaze perception task, respectively: Continuous confidence ratings were regressed on the fixed-effect predictors accuracy, AQ score, and their interaction. The table presents fixed-effect parameter estimates *β* (with standard error of the mean, SEM, in brackets), t-statistic, degrees of freedom (df), and *p*-value. ∗*p* < 0.05, ∗∗*p* < 0.01, and ∗∗∗*p* < 0.001.

Given that accuracy in the implicit task was driven by body-head congruency and gaze validity, we ran an additional model to control for whether these subtle social cues could potentially affect confidence ratings in relation to autistic traits. Our results show, as expected, that participants were more confident in the body-incongruent, gaze-valid condition compared to the body-congruent condition (gaze validity x body-head congruency: *β* = −0.37, *z* = −5.39, *p* < 0.001), in line with their accuracy in the implicit task. However, AQ scores did not significantly affect these confidence ratings (gaze validity x body-head congruency x AQ scores: *β* = −0.07, *z* = −1.06, *p =* 0.29), suggesting that autistic traits did not influence performance in the implicit task, nor did they modulate confidence based on task accuracy.

Finally, we tested the fourth prediction that autistic traits modulate metacognitive sensitivity in both explicit and implicit gaze perception. To quantify metacognitive sensitivity, we calculated the area under the type 2 ROC curve (AUROC2) for each participant, reflecting how accurately participants’ confidence ratings corresponded to their performance in relation to autistic traits (AQ scores). Five participants in the explicit and three participants in the implicit task were outliers according to the Mahalanobis distance measure,^39^^,^^40^ and thus were excluded from the correlation analysis between autistic traits and AUROC2 scores. We computed a nonparametric correlation (Spearman) to compare AUROC2 scores with AQ scores.

In the explicit task, a significant negative correlation was observed between autistic traits and metacognitive sensitivity (AUROC2), with higher AQ scores associated with reduced metacognitive sensitivity (*r* = −0.34, *t*(59) = −2.73, *p* < 0.01; Figure 5E). Even when including outliers, a significant correlation between AUROC2 scores and autistic traits was observed (*r* = −0.30, *t*(64) = −2.51, *p* = 0.01), supporting the robustness of the effect. Further, we performed a post hoc analysis (*t* test) using a median split on AQ scores (median = 16), which demonstrated that participants with higher AQ scores showed significantly lower metacognitive sensitivity (AUROC2 = 0.44) than those with lower AQ scores (AUROC2 = 0.54; t = −1.71, *p* = 0.05). In the implicit task, however, we found no significant correlation between AQ scores and metacognitive sensitivity (*r* = 0.01, *t*(61) = −0.06, *p* = 0.95; Figure 5F). These findings suggest that metacognitive sensitivity is reduced in individuals with higher autistic traits during explicit gaze perception but remains unaffected in implicit gaze processing.

## Discussion

The main goal of this study was to systematically investigate whether autistic traits distinctly modulate implicit and explicit gaze perception as well as their metacognitive representations. To achieve this, we used the same real-life images featuring an actor gazing at laterally presented target objects, based on which participants performed a gaze judgment task and an object detection task quantifying explicit and implicit gaze perception. Our findings reveal a dissociation between implicit and explicit gaze processing, demonstrating that they are differently affected depending on the degree of autistic traits. Implicit gaze perception ability did not modulate performance in the explicit gaze perception task, suggesting that these two processes rely on distinct mechanisms. Importantly, individuals with stronger autistic traits experienced difficulties in explicitly inferring others’ attentional focus, while their implicit perception of others’ attentional focus remained unaffected. These deficits in explicit gaze processing were associated with reduced metacognitive sensitivity. Together, our results provide insights into the complex interplay between implicit and explicit gaze perception, their metacognitive representations, and how this interaction depends on autistic traits.

Our results demonstrate that viewing another person whose body is facing straight ahead but with the head rotated toward the side, which implicitly signifies that something has caught that person’s attention, facilitates attention orienting. This finding extends previous results, showing that body-head incongruency leads to faster reaction times in detecting a laterally presented object.^18^ Thus, humans implicitly integrate a range of social cues to facilitate attentional shifts, suggesting that reorienting one’s attention is driven by more complex social cues than merely following gaze direction. This finding aligns with previous work showing that our brains automatically and implicitly construct a rich model of others’ attention that goes far beyond simply reconstructing others’ gaze direction,^41^^,^^42^ facilitating the prediction of others’ attention.^43^

The same attentional mechanisms that automatically reorient our attention toward the saliency of others’ gaze may drive deliberate, conscious representation of the other's attentional focus. This is consistent with previous evidence suggesting that unconscious gaze perception shapes how we interpret others’ attentional states.^10^^,^^11^^,^^44^ However, orientation of the body with respect to the head did not affect participants’ performance in our explicit gaze perception task, suggesting that the combination of social cues such as gaze, head, and body orientation modulated implicit attentional shifts, while, in contrast, explicit judgments of others’ gaze were unaffected. Crucially, performance in implicit gaze perception did not predict performance in explicit gaze perception, suggesting that automatic attentional shifts driven by perceived saliency of others’ social cues do not facilitate explicit perception of others’ attentive gaze. Together, these findings suggest that the mechanisms underlying implicit and explicit perception of others’ gaze are relatively distinct, contradicting the notion that the two processes share an underlying social mechanism.

Stronger autistic traits significantly predicted worse gaze perception accuracy in the explicit task, as predicted. However, implicit processing of gaze cues was not modulated by autistic traits, contrary to our prediction. In contrast to prior findings suggesting that individuals with ASD struggle to shift their attention based on incongruent body-head orientation,^18^ our results indicate that individuals with strong autistic traits were able to perceive the saliency of others’ gaze cues implicitly. Our findings are thus more compatible with previous work suggesting that individuals with ASD implicitly process gaze cues in a similar manner as neurotypicals.^45^^,^^46^^,^^47^ Nevertheless, there is emerging evidence that, in contrast to neurotypicals, ASD individuals may use different strategies relying more strongly on perceptual features of gaze cues,^47^^,^^48^^,^^49^^,^^50^ while the social meaning represented by social cues is not fully understood.^51^^,^^52^^,^^53^ Thus, individuals with higher autistic traits may rely on different cognitive strategies to implicitly process subtle social cues, such as body-head orientation, to guide attention. Crucially, our results show that explicit, but not implicit, gaze processing mechanisms are affected by autistic traits. Our findings demonstrate a dissociation in metacognitive sensitivity between explicit versus implicit processing of others’ gaze with respect to autistic traits. As predicted, participants with stronger autistic traits showed significantly reduced metacognitive sensitivity for explicit gaze perception judgments, which aligns with the notion that difficulties in representing one’s own mental states (metacognition) in autism are intimately coupled with difficulties in representing others’ mental states (mentalization).^35^^,^^36^ Our results are in line with theoretical accounts proposing that mentalization and metacognition rely on shared underlying systems, which are similarly affected in autism.^33^^,^^34^ Our findings are also compatible with previous studies showing that individuals with autism or high autistic traits often face difficulties in complex social inference tasks that require explicit reasoning.^54^^,^^55^^,^^56^^,^^57^ However, contrary to our prediction, participants with stronger autistic traits showed no reduction in metacognitive sensitivity in the object detection task involving implicit gaze processing; again, supporting the notion that the mechanisms underlying implicit and explicit perception of others’ gaze are relatively distinct.

Overall, our findings paint a complex picture of the relationship between gaze perception, metacognition, and autism, where autistic traits affect metacognitive abilities differently in explicit and implicit gaze processing. Our results may help explain some of the inconsistencies between previous studies on metacognition, ToM, and autism, where some studies have found that autistic individuals experience difficulties in metacognition alongside their ToM deficits,^29^^,^^30^^,^^36^ while others observed no association between impairments in ToM and metacognitive abilities.^58^^,^^59^ We propose that quantifying implicit and explicit perceptual processes in the same individuals using identical sets of stimuli may provide a promising experimental approach for revealing the link between mentalizing and metacognition.

### Future directions

Future research could include additional methods, such as eye-tracking, for a follow-up to explain some of the differences between implicit and explicit gaze perception in more detail. One could examine fixation patterns on the actor’s head, body, and objects in implicit versus explicit tasks to determine whether attentional strategies differ across tasks and as a function of autistic traits. In particular, individuals with higher autistic traits may show stronger object-focused fixations, whereas individuals with lower autistic traits may show stronger gaze-focused fixations.

### Conclusions

Our findings suggest a dissociation between implicit and explicit gaze perception with respect to the degree of autistic traits. Automatic, stimulus-driven shifts in participants’ attention induced by social gaze cues remained intact across varying degrees of autistic traits, while explicit gaze perception ability decreased with stronger autistic traits. Moreover, our study demonstrates that individuals with higher autistic traits not only show difficulties in explicitly constructing others’ attention, but also show reduced metacognitive sensitivity to these challenges. This pattern of results suggests disruptions in both the explicit reconstruction of others’ attention and in the self-monitoring processes supporting these processes.

### Limitations of the study

Our findings on implicit gaze perception indicate that individuals with strong autistic traits are able to shift their attention based on incongruent body-head orientation equally well as individuals with weak autistic traits, contrary to prior evidence.^18^ This discrepancy between the results could potentially be explained as Ashwin (2015) focused on reaction times as outcome measures, which are arguably more sensitive for detecting subtle between-condition differences. Furthermore, Ashwin and colleagues tested participants diagnosed with autism versus neurotypical controls, which should be associated with greater effect sizes compared to studying neurotypical subjects with varying degrees of autistic traits. Differences between individuals with higher versus lower autistic traits may have been too subtle to be detected using accuracy-based outcome measures in the implicit task.

Another limitation of the present design concerns differences in stimulus presentation time and task demands between the implicit and explicit gaze perception tasks, which may have influenced how participants perceived the actor's gaze in these separate tasks. In the implicit task, participants were exposed to the real-life images for a shorter duration (450 ms) than in the explicit task (2 s). We designed the implicit task by presenting a fixation cross followed by a very short presentation of a real-life image to assess the automatic reorienting of attention towards the actor's subtle social signals. Thus, in the implicit task, participants' attention was strongly guided by the initial focus on the actor's gaze, head, and body orientation. In contrast, in the explicit task, participants were longer exposed to the stimuli, thus able to move their attention to different parts of the image scene, such as the actor's gaze or the objects, to perform the task. These differences may partly explain why effects observed in the implicit task did not translate directly to explicit gaze judgments.

## Resource availability

### Lead contact

Further information and requests for resources should be directed to and will be fulfilled by the lead contact Patricia Christian: patricia.christian@ki.se.

### Materials availability

All images (real-life photographs) created for the implicit and explicit experiments are available upon request by contacting the lead contact.

### Data and code availability

• Data/Code: The data and code used for the analysis have been deposited on an OSF repository (https://osf.io/t9hps/?view_only=23b6942741af45fb9e4c7d65bb0a70eb). They are publicly available as of the date of publication.
• Additional information: Any additional information required to reanalyze the data reported in this paper is available from the lead contact upon request.

## Acknowledgments

We thank Beatrize Widlund for contributing to the data collection. This work was supported by the 10.13039/501100000781European Research Council (101220685-MINDSIM), the 10.13039/501100004359Swedish Research Council (2021-02089), the Swedish Foundation for Strategic Research (FFL21-0261), the 10.13039/100014437Wenner-Gren Foundations (FT2021-0003), the 10.13039/501100003792Swedish Brain Foundation (FO2024-0385), the 10.13039/501100007687Swedish Society of Medicine (SLS-960489), the 10.13039/501100003748Swedish Society for Medical Research (10.13039/501100003748SSMF) (SG-24-0184-B-H-01), and the 10.13039/501100011898Marcus and Amalia Wallenberg Foundation (2024.0042).

## Author contributions

P.C. and A.G. conceptualized the idea and design of the study. P.C. and R.L. developed the task stimuli, and R.L. performed data collection. P.C. and A.G. analyzed data; P.C., R.L., and A.G. wrote the manuscript.

## Declaration of interests

The authors declare no competing interests.

## STAR★Methods

### Key resources table

**Table:** 

REAGENT or RESOURCE	SOURCE	IDENTIFIER
**Deposited data**		

Raw Data and Code	This paper	OSF: https://osf.io/t9hps/?view_only=23b6942741af45fb9e4c7d65bb0a70eb

**Software and algorithms**		

R 4.4.1	R Core Team	https://www.r-project.org, RRID:SCR_001905
lme4	CRAN	https://cran.r-project.org/web/packages/lme4/index.html, RRID:SCR_015654
psych	CRAN	https://CRAN.R-project.org/package=psych, RRID:SCR_021744
stats	CRAN	https://cran.r-project.org/doc/manuals/r-patched/packages/stats/refman/stats.html, RRID:SCR_025678

### Experimental model and study participant details

Sixty-four healthy volunteers (mean age = 31 years, range = 19-59 years; 38 females) took part in the study. All participants were recruited at the Karolinska Institutet and had no known psychiatric or neurological disorders. They were all naïve with respect to the aims and hypotheses of the study. An *a priori* power analysis (GPower) based on the effect size of Cohen’s d = 0.58 reported in a previous study on naturalistic gaze perception suggested that 63 participants are sufficient to detect significant effects (α = 5%) with a power of 90%.^60^ Ethical approval was granted by the Swedish Ethical Review Authority (Ethical approval number: 2023-01330-01) and the procedures followed the principles of the Declaration of Helsinki. All participants gave their written informed consent prior to participation and received a monetary compensation of €10.

### Method details

#### Experimental procedures

Participants were positioned 62 cm from the display and a chin rest was used for head stabilization. The visual stimuli were displayed on a Dell Optiplex 7040 display with a 1,920 × 1,080-pixel image resolution and a refresh rate of 60 Hz. Participants completed the two experimental tasks in a fixed order: they first performed the object detection task (implicit gaze perception), followed by the gaze judgment task (explicit gaze perception). This fixed order minimized the risk that participants in the implicit task would focus on perceiving the actor’s gaze direction, which was the goal of the explicit task. Nevertheless, to control that participants did not focus on the actor’s gaze and did not infer that gaze direction was relevant for task performance, we used a debriefing questionnaire after completion of the implicit task. In this questionnaire, participants were asked what they believed the goal of the task was and which aspects of the scene they focused on during task performance. Critically, after completing the implicit task, none of the participants reported that they believed the goal of the experiment was related to social stimuli (actor’s gaze) presented in the scene, nor did any report focusing on the actor’s gaze during the task, or that the actor’s gaze affected their responses. For each task, participants were given written instructions on the computer screen and, if necessary, verbal clarifications by a research assistant. Participants performed 10 practice trials before starting each experiment.

#### Experimental stimuli

The experimental stimuli consisted of 96 real-life images (1960 × 1080 pixels) featuring an actor with a neutral facial expression positioned centrally between two tables, each displaying four different objects. The actor’s gaze was directed at one of the objects (target object). The objects were placed at four different locations at each table. Each object position on one table mirrored its corresponding object position on the opposite table, creating eight object positions for each image. Specifically, each side displayed two objects at standard tabletop height and two elevated by 20 cm using a box or tray. The objects at the same height were positioned 20 cm apart from each other. Altogether, 24 different objects in total were used, each randomly presented at various positions within the images. Throughout the experiments, each object was presented once as the gazed-at object and once in an equivalent position on the contralateral side in a counterbalanced randomized order. The actor’s upper body and head were fully visible in all images. Similar to a previous task design,^13^^,^^18^ the head position was oriented 35 degrees to the side while the body was either aligned at the same 35-degree angle or directed straight towards the camera, creating two conditions: In the body-head congruent condition, both the head and body were oriented 35 degrees to the side, while in the body-head incongruent condition, the head was oriented at a 35-degree angle to the side and the body faced straight forward (Figure 1E). Half of the trials were presented in the body-head congruent condition, while the other half were displayed in the body-head incongruent condition, in a randomized order. The same real-life images were used in the explicit and implicit gaze perception tasks. By using the same images in both tasks, the impact of body-head orientation on task performance could be assessed in both implicit and explicit gaze perception.

#### Explicit gaze perception task

Participants performed a gaze target detection task similar to previous paradigms in which participants were instructed to indicate which object the actor was gazing at.^20^^,^^24^^,^^25^ In each trial, the actor gazed at one out of four objects positioned on the left or right table relative to the actor (Figure 1C). Each real-life image was displayed for 2 s to limit excessive visual scanning, followed by a response window where four objects were presented (Figure 1A). In a forced-choice task, participants had to select which of the four objects the actor was gazing at, using a button press, without any time constraint. Next, participants were asked to rate their confidence in their decision (no time constraint) on a continuous scale ranging from 0 to 100, in steps of 10, where 0 indicated “Not sure at all” and 100 indicated “Very sure.” This confidence rating provided a measure of metacognitive sensitivity, reflecting the participants’ ability to monitor and evaluate their own task performance on a trial-by-trial basis.^31^^,^^61^ Note that we did not provide any performance feedback in either the explicit or the implicit task, which might have biased the confidence ratings.

On each trial, the actor looked left or right at one of the four possible object positions on the table, and the actor’s body-head orientation was either congruent or incongruent, generating 16 trial types. All trial types were balanced and presented in a random order. For analysis, trial types were collapsed into two major conditions: body-head congruent and body-head incongruent. Subjects performed 96 trials in 8 blocks of 12 trials each, thus 48 trials per major condition.

#### Implicit gaze perception task

For the implicit task the real-life images were designed as a naturalistic gaze-cueing paradigm, inspired by a previous study.^38^ Here, participants performed a visual object detection task in which they were instructed to detect the presence of an object among the eight presented objects in the scene. The cued (to be detected) object was either congruent with the actor’s gaze (gazed-valid) or incongruent with the actor’s gaze (gaze invalid). Crucially, the actor’s gaze direction and head and body orientations were task-irrelevant, as participants were instructed to perform an object detection task. Gaze-cuing paradigms have been characterized by faster attentional orienting toward gazed-at targets compared to non–gazed-at targets, reflecting an automatic shift of attention in the direction of another individual’s gaze. We used this experimental design to study implicit, automatic allocation of attention driven by the actors gaze direction.

To ensure that participants were familiar with all target objects, before the testing started, they were presented with a list of all 24 objects (object names) that they would need to detect later in the images. They were encouraged to ask questions if any items were unfamiliar or if they struggled to visualize how the objects would look like. In such cases, the research assistant provided additional descriptions to ensure that participants could reliably identify all 24 objects in the real-life images.

The structure of one trial is shown in Figure 1B. Each trial began with displaying the object’s name that the participant should look for in the subsequent image (for 1 s), followed by a fixation cross for 500 ms. The fixation cross coincided with the location of the actor’s chin to ensure that the actor’s head, gaze, and body orientation were clearly visible to the participants upon image onset.^13^ The same real-life images as in the explicit task featuring an agent gazing at one of 8 displayed objects (Figure 1D) were then presented for 450 ms. The rationale for using this relatively brief duration was to make the task sufficiently difficult to avoid performance ceiling effects. This specific duration was determined based on piloting and previous studies showing that participants need on average 450 ms to detect a gaze-valid object following a gaze cue,^18^ which is consistent with work demonstrating that gaze cueing of attention typically emerges within this time frame.^9^ After image presentation, participants were asked to indicate whether they believed the cued object was present in the image or not (forced choice, no time limit) by pressing the left arrow key for “Yes” and the right arrow key for “No”. The position of the response options, appearing on the left or right side of the screen, was counterbalanced across trials to prevent potential response bias. Following this forced choice, participants were asked to rate confidence in their response using the same continuous rating scale as in the explicit gaze perception task (see above).

On each trial, the pre-specified object was either present or absent, the actor’s body-head orientation was either congruent or incongruent, and the actor was either looking left or right, at one of the four possible object positions on the table, generating 32 trial types. All trial types were balanced and presented in a randomized order. When the cued object was present (i.e., in 50% of the trials), the actor was either gazing at the pre-specified object (gaze-valid) or in the direction opposite to the cued object (gaze-invalid). For analysis, we only considered the trials in which cued object was present in the image, and collapsed trial types into four main conditions: gaze-valid + body-head congruent, gaze-valid + body-head incongruent, gaze-invalid + body-head congruent, and gaze-invalid + body-head incongruent. Subjects performed 96 trials in 8 blocks of 12 trials each, thus 12 trials per major analysis condition.

#### Post-experiment questionnaires

After concluding the experimental testing, participants filled out the AQ questionnaire. The AQ questionnaire is a self-report instrument to quantify traits associated with autism spectrum disorder (ASD), which has been shown to have a high test-retest and inter-rater reliability (Baron-Cohen et al., 2001). The questionnaire consists of 50 items in total and responses are indicated using a 4-point Likert scale. The AQ score reflects the degree of autistic traits, with higher scores indicating stronger autistic traits. In this study, AQ scores ranged from 2 to 27 (*M* = 16.1, *SD* = 6.08, see Figure 4C, with no participants exceeding the clinical cut-off of 32 points.

### Quantification and statistical analysis

#### Implicit and explicit gaze perception

We analyzed effects on task performance (accuracy) in implicit and explicit gaze perception using generalized linear mixed models (GLMMs) implemented in the lme4 package in R.^62^ The alpha threshold was set at 5% (two-tailed) for all analyses.

First, we tested the prediction that incongruent body-head congruency facilitates performance in the implicit, but not explicit gaze perception task. In the first step, we tested whether implicit gaze perception was modulated by body-head congruency: we computed a GLMM model in which binary accuracy (0 = incorrect, 1 = correct) was regressed on fixed-effect predictors for gaze validity (0 = invalid gaze cue, 1 = valid gaze cue), body-head congruency (0 = body-head incongruent, 1 = body-head congruent), and their interaction term for the implicit gaze perception task. All fixed-effect predictors were also modeled as random slopes in addition to participant-specific random intercepts. Continuous predictors were z-transformed. To assess participants’ ability to detect objects in the implicit gaze perception task, our analysis included only trials in which the object was present, thus half of all trials. In the next step we tested whether body-head congruency similarly modulates explicit gaze perception as implicit understanding of others’ gaze. We calculated an additional GLMM in which accuracy in the explicit gaze perception task was regressed on the fixed-effect predictor of body-head congruency. Since all gaze cues in the explicit task were valid, this model did not include gaze validity as a fixed-effect predictor.

To test the second hypothesis, we extracted individual coefficients (individual fixed-effects estimates of gaze validity × body-head congruency interaction) from the implicit GLMM model, representing individual attention shifting towards others’ salient gaze cues. These individual estimates were then included as a fixed-effect predictor (implicit_accuracy) in the explicit GLMM to assess whether individual differences in implicit gaze performance predict accuracy in explicit gaze perception. In this explicit GLMM model, binary accuracy in the explicit task (0 = incorrect, 1 = correct) was regressed on the fixed-effect predictor implicit_accuracy and their interaction term.

Third, we tested whether individual differences in autistic traits modulated implicit and explicit gaze perception performance. We tested whether implicit and explicit gaze accuracy changed as a function of autistic traits by including continuous AQ scores as a fixed-effect predictor to the respective GLMM for implicit and explicit gaze perception. Higher AQ scores indicated higher levels of autistic traits. This allowed us to assess whether higher levels of autistic traits were associated with reduced accuracy in either task.

#### Metacognitive representation of implicit and explicit gaze perception

Metacognition and metacognitive sensitivity were calculated following established frameworks for modeling the direct link between confidence and accuracy, established in prior studies.^31^^,^^61^ Here, we tested whether autistic traits modulate confidence ratings in the implicit or explicit gaze perception task. For this purpose, we computed linear mixed models (LMMs) in which confidence (continuous rating scale) was regressed on fixed-effect predictors for accuracy (0 = incorrect, 1 = correct), AQ score and their interaction term for the explicit and implicit task, respectively. Additionally, for the implicit task, we ran a linear mixed-effects regression (LMER) model on confidence ratings, using similar fixed-effect predictors as in the GLMER accuracy models, including gaze validity (0 = invalid gaze cue, 1 = valid gaze cue), body-head congruency (0 = body incongruent, 1 = body congruent), AQ score, and their interaction terms. Hereby, we could test whether these subtle social cues such as gaze, head and body orientation could potentially affect confidence ratings in relation to autistic traits.

Next, we tested for metacognitive sensitivity – i.e., how well an individual’s confidence ratings could distinguish between accurate and inaccurate task performance – we calculated the hit and false alarm rate pairs with increasing confidence levels for each subject. The area under the Type 2 Receiver Operating Characteristic curve (AUROC2) is often used as an index of metacognitive sensitivity, reflecting the ability to monitor and evaluate one’s own cognitive performance.^31^ To calculate AUROC2 each confidence level was taken as a criterion to differentiate between low and high confidence trials. Confidence was rated on an 11-point Likert scale. Beginning with a criterion where only a rating of zero indicated low confidence and any higher rating indicated high confidence, we gradually raised the criterion until all ratings below the maximum score (100) were considered low confidence, with only the highest rating classified as high confidence. For each confidence level we plotted the resulting probabilities for hits, p (high confidence|correct), and false alarms, p (high confidence|incorrect) against each other. Higher AUROC2 values indicate better metacognitive sensitivity, while lower individual data points indicating a lower ability to accurately assess the certainty of one’s own decisions, indicating impaired metacognitive performance. To explore whether metacognitive sensitivity was linked with autistic traits, we calculated a non-parametric Spearman correlation based on previous approaches.^35^
